# Exosomal delivery of doxorubicin enables rapid cell entry and enhanced *in vitro* potency

**DOI:** 10.1371/journal.pone.0214545

**Published:** 2019-03-29

**Authors:** Christina Schindler, Andie Collinson, Carl Matthews, Amy Pointon, Lesley Jenkinson, Ralph R. Minter, Tristan J. Vaughan, Natalie J. Tigue

**Affiliations:** 1 Department of Antibody Discovery and Protein Engineering, MedImmune Ltd., Cambridge, United Kingdom; 2 Mechanistic Safety and ADME Sciences, Drug Safety and Metabolism, IMED Biotech Unit, AstraZeneca, Cambridge, United Kingdom; Brandeis University, UNITED STATES

## Abstract

Doxorubicin is a chemotherapeutic agent that is commonly used to treat a broad range of cancers. However, significant cardiotoxicity, associated with prolonged exposure to doxorubicin, limits its continued therapeutic use. One strategy to prevent the uptake of doxorubicin into cardiac cells is the encapsulation of the drug to prevent non-specific uptake and also to improve the drugs’ pharmacokinetic properties. Although encapsulated forms of doxorubicin limit the cardiotoxicity observed, they are not without their own liabilities as an increased amount of drug is deposited in the skin where liposomal doxorubicin can cause palmar-plantar erythrodysesthesia. Exosomes are small endogenous extracellular vesicles, that transfer bioactive material from one cell to another, and are considered attractive drug delivery vehicles due to their natural origin. In this study, we generated doxorubicin-loaded exosomes and demonstrate their rapid cellular uptake and re-distribution of doxorubicin from endosomes to the cytoplasm and nucleus resulting in enhanced potency in a number of cultured and primary cell lines when compared to free doxorubicin and liposomal formulations of doxorubicin. In contrast to other delivery methods for doxorubicin, exosomes do not accumulate in the heart, thereby providing potential for limiting the cardiac side effects and improved therapeutic index.

## Introduction

Doxorubicin is a highly effective anthracycline antibiotic that has been used for over four decades in the treatment of a broad range of cancers, such as hematological malignancies and solid tumours [1]. Despite its potent anti-tumour properties, patients treated with doxorubicin can experience side-effects, the most severe being cardiomyopathy leading to congestive heart failure [2, 3]; a phenomenon that is dependent upon, and thus limits, the cumulative dose. Deciphering the mechanisms of doxorubicin-induced cardiotoxicity remains an unresolved and intensely studied area of research. There are, however, two organelles that have received the most attention with respect to their role in doxorubicin activity; the nucleus and the mitochondria. Once transported into the nucleus it is believed that doxorubicin exerts its effects via a number of mechanisms including intercalation of DNA [4] and the binding to proteins involved in DNA replication and transcription [5]. In the mitochondria, proposed mechanisms include binding to mitochondrial DNA [6] and the production of reactive oxygen species (ROS) causing oxidative stress [7].

Strategies to minimise this toxicity and increase the therapeutic window of doxorubicin have been developed and include prolonged infusion vs bolus dosing [8], co-administration with cardioprotective agents such as dexrazoxane, an iron chelator that protects cells from mitochondrial ROS production [9], and encapsulation of doxorubicin in drug delivery vehicles such as nanoparticles, hydrogels and liposomes [10]. Two formulations of liposomal doxorubicin have been approved for clinical use: PEGylated liposomal doxorubicin (PLD) such as Doxil/Caelyx, and non-pegylated liposomal doxorubicin (NPLD) (Myocet). Encapsulation of doxorubicin reduces the amount of free drug in the circulation and prevents the uptake into cells via passive diffusion. Due to their size, liposomal formulations avoid renal clearance, thereby increasing the circulatory half-life of the encapsulated drug. PLD is smaller than NLPD (80 vs 180 nm diameter) and can more readily penetrate the leaky tumour vasculature via the enhanced permeability and retention (EPR) effect [11, 12]. Due to its PEGylated coat it also avoids detection by the mononuclear phagocytic system, thereby increasing the circulation time in the blood. As a result this, however, liposomal doxorubicin can accumulate in the skin and cause side effects such as palmar-plantar erythrodysesthesia, commonly known as hand-foot syndrome [13, 14].

Exosomes are naturally occurring extracellular vesicles and represent an attractive alternative delivery vehicle for chemotherapeutic drugs. These small (approx. 80 nm) vesicles, containing selected RNA and protein content of the host cell, are released and can efficiently deliver their bioactive cargo to recipient cells via several active transport pathways; the preferred internalisation routes often being different between different cell-types [15–19]. In addition to carrying their natural content, it has been shown that exosomes can be manipulated to enable loading with small molecule therapeutics [20–22]. From recent studies, it has emerged that the potency of doxorubicin encapsulated within exosomes or exosome-like vesicles, can either match, or even surpass the potency of the free drug and/or liposomal formulations [22–24]. We hypothesised that the increased potency of exosome encapsulated doxorubicin can in part be explained by the highly efficient cellular uptake mechanisms for exosomes. In contrast to most previous studies on exosome-encapsuled small molecule drugs that report data for a single dose and timepoint, we performed a titration and timecourse analysis with a focus on the initial uptake of doxorubicin-loaded exosomes. We indeed found that exosomal doxorubicin is taken up faster into recipient cells than free or liposomal doxorubicin. Following uptake, exosomal doxorubicin is rapidly released from endocytic compartments and accumulates to higher intracellular levels compared to free or liposomal doxorubicin. As a consequence, we observed the onset of mitochondrial swelling followed by apoptotic cell death within hours of cell treatment. By exploiting the natural tissue tropism of exosomes compared to liposomes or the free drug, or by adopting targeting approaches, exosome-based formulations could present novel highly efficient drug delivery systems for doxorubicin and potentially more broadly to enable an improved therapeutic index.

## Experimental prodedures

### Cell Culture

The HEK293 cell line was purchased from the European collection of authenticated cell cultures (ECACC), BT-20 and SK-BR-3 cell lines were acquired from the American Type Culture Collection (ATCC). HEK293 and BT-20 cells were grown in MEM (ThermoFisher Scientific) supplemented with 10% FBS and 1% Non-essential amino acids (NEAA; Thermo Fisher Scientific). SK-BR-3 cells were propagated in Mc Coy’s medium (Thermo Fisher Scientific) supplemented with 10% FBS. Primary pulmonary artery smooth muscle cells (PASMC) and human umbilical vein endothelial cells (HUVEC) were purchased from Lonza and grown in SmBM or EBM-2 medium (Lonza), respectively and used at passage 2 (PASMC) or passage 3 (HUVEC). hiPS-derived cardiomyocytes were purchased from FUJIFILM Cellular Dynamics and grown in the supplied medium according to manufacturer’s instructions and were used in experiments on day 9/10 in culture. All cells were maintained in 5% CO2 at 37°C and determined to be negative for mycoplasm in routine testing.

### Exosome isolation and quantification

For exosome isolation, adherent HEK293 cells were rinsed twice in PBS and then incubated serum-free Freestyle medium (ThermoFisher Scientific). After 3 days, conditioned medium was collected and exosomes were isolated by filtration as described in [25]. During the isolation, the buffer was exchanged to PBS. For comparison, the conditioned medium was processed by centrifugation for 10 min at 1000xg to remove cells and large debris, followed by 20 min at 10,000xg in an SS34 rotor (Sorvall). Pellets were discarded and supernatants further processed by ultracentrifugation at 110,000xg for 75 min using a TLA-45 rotor (Beckman). Supernatants were discarded and exosome-containing pellets were resuspended in a volume of PBS corresponding to ~1/250 of the original volume. Nanoparticle tracking on a NS300 system (Malvern) and the NTA3.0 software was utilized to determine the concentration of exosomes as well as their mean size and size distribution with the same instrument and software settings used for all samples. Settings were as follows: 5 movies of 60 second duration at room temperature were acquired for each sample at camera level 15. The processing threshold was kept constant (at 6) for all measurements. NTA3.0 software was used to determine average particle size and count and mode sizes of 90-120nm in diameter were obtained for exosome samples independent of isolation method. Exosome purity, as indicated by a particles/μg protein value [26], was determined by dividing the particle count by total protein concentration as measured using a micro BCA protein assay kit (Thermo Fisher Scientific)).

### Electroporation loading of doxorubicin

5e^10^ exosomes were diluted in PBS to a volume of 200 μl, doxorubicin (Sigma-Aldrich) was added at 1.25 mM followed by addition of an equal volume of cold 2x electroporation buffer (PBS, 600 mM sucrose). Electroporation was carried out in 4 mm cuvettes in a Gene Pulser II Electroporator (BioRad) at 250V and 125 μF. Exosomes were recovered for 20 min at 37 °C. Unincorporated doxorubicin was removed by 3 consecutive spin desalting columns (ThermoFisher Scientific) in which also the sucrose concentration in the sample was stepwise lowered to 150 mM, 50 mM and 10 mM in the last desalting step. The concentration of encapsulated doxorubicin was determined using the intrinsic fluorescence of doxorubicin on a Quantus Fluorimeter (Promega) with settings ex: 495 nm SP, em: 510–580 nm.

### Exosome uptake assay

HEK293 cells were seeded in 96 well collagen coated plates (GreinerBioOne) in MEM, 10% FBS and 1% NEAA for 2 days at a density of 2e^4^ cells/well. For the assay, cells were washed once with Freestyle medium and 75 μl/well Freestyle medium was added followed by 25 μl of 4-fold serial dilutions of doxorubicin, Myocet (TEVA), Doxil or control liposomes (Avanti Polar lipids) in PBS with 1.1e^-4^ M (64 μg/ml) being the highest concentration tested. Alternatively, 25 μl doxorubicin-loaded exosomes were added at 2-fold serial dilution in PBS. After incubation at 37 °C 5% CO2 for times as indicated cells were washed twice with PBS and processed for analysis. For flow cytometry cells were dissociated with accutase (Thermo Fisher Scientific) and washed 3 times in PBS supplemented with 2% FBS. Doxorubicin content was analysed on a Fortessa (BD) using TexasRed-PE filter settings. Live cells were defined by DAPI-exclusion. Data was analysed using the FloJo version 10 software (TreeStar). For comparison between samples the geometric mean of the medium fluorescence instensity (MFI) was determined. For microscopy Hoechst33342 (ThermoFisher Scientific) was added at 1 μg/ml for 10 min. 2 images per well were acquired at 20x magnification on an image express high content epifluorescence microscope (Molecular Devices) using filter settings for DAPI and Cy3.

### Time-lapse video microscopy

HEK293 and PASMC cells were seeded at 1e^4^ or 2.5e^3^ per well in collagen coated 96 well plates. After 48 h medium was replaced with 75 μl fresh complete medium containing Caspase 3 sensitive substrate DEVD-nucview488 (Biotinum) or Mitotracker green (ThermoFisher Scientific) and subsequently 25 μl of Dox-exosomes or Dox at concentrations indicated in the figure legend was added. Final assay concentrations were 1x Caspase 3 substrate and 50 nM Mitotracker green, which were established to not affect cell growth in a preliminary experiment. Cells were imaged every 2h for 48h in an IncucyteZoom (EssenBioScience) using a 20x objective. At least 2 images per well and timepoint were analysed. Processing definitions were created in the acompaning software to determine phase and fluorescence confluency (in % image area). To exclude effects from slighly different seeding densities or cell death during the experiment, fluorescence confluency was divided by phase confluency for analysis.

### Viability assay

HEK293, SK-BR-3, BT20, PASMC, and HUVEC were seeded in 384-well collagen coated plates (GreinerBioOne) in 30 ul of medium at a density of 1e^3^ cells/well. Seeding density for iPS cardiomyocytes was 5e^3^ cells/well. For the assay, 10 μl of 4-fold serial dilutions of doxorubicin, Myocet, Doxil or control liposomes were added (11 sample points, dilutions were in PBS). Alternatively, 10 μl of 4-fold serially diluted doxorubicin-loaded exosomes were added. Cells treated with an equal volume of PBS served as control. After 48 h, ATP content indicating viable cells was determined using the Cell Titer glo assay (Promega) according to manufacturer’s instructions. The same assay was used in PASMC, HUVEC and icell cardiomyoctes with the exception that the readout using Cell titer glo was performed 72 h after addition of the drugs.

### Statistical analysis

All data from analysis of cellular uptake is presented as mean +/- SD from n = 3 independent experiments. Statistical analysis was performed in GraphPadPrism 6 (GraphPad Inc.) using a one-way ANOVA followed by a multiple comparisons test. P-values shown represent * p<0.05 and **** p<0.0001. IC_50_ values for the potency of doxorubicin, Exo-Dox and liposomal formulations were obtained in GraphPadPrism from plotting the logarithm of concentration in X and RLU (Relative Luminescence Units) in Y followed by a 4-parameter logistic equation to give IC_50_ values. Y = Bottom + (Top-Bottom) / (1+10^((LogIC_50_-X)*HillSlope)). Data is presented as mean +/- SD from n = 3 independent experiments unless stated otherwise in the figure legends. Data from timelapse videomicroscopy is presented as mean +/- SEM.

## Results

### Isolation of extracellular vesicles and loading with doxorubicin

For production of extracellular vesicles (EV), HEK293 cells were cultured in enriched serum-free medium; cell viability was >90% over the course of the 3 day culture (S1a Fig). EV were prepared from the conditioned media using a filtration method described in Longatti et al. [25]. To validate this convenient and scalable method of purification, the EV were analysed by Western blotting and nanoparticle tracking analysis (NTA) and compared to EV purified by the traditional ultracentrifugation method [27]. Western Blot analysis of the EV purified by both methods demonstrated an enrichment of EV markers CD63, Alix and Tsg101 compared to the cell lysate and absence of the endoplasmic reticulum resident protein calnexin, a marker for cellular debris; fibronectin, which serves as a marker for extracellular matrix, was present at the same level irrespective of the preparation method (S1b Fig) [28]. Purity was assessed as ratio of particle number over protein content (S1c Fig) [29]. Nanoparticle tracking analysis (NTA) demonstrated a narrow size distribution with a mean diameter of ~75 nm for both EV preparations (S1d Fig). These data illustrate that vesicle populations isolated from both purification processes have properties consistent with exosomes for this reason, we will refer to the isolated vesicles as exosomes.

Loading of exosomes with Dox via electroporation was optimised by varying the exosome and doxorubicin (Dox) concentrations, as well as electroporation and buffer conditions using the intrinsic red fluorescence to monitor incorporation. Typical doxorubicin loading ranged between 1 and 1.5 μg/ml Dox; Dox loading efficieny is depicted in S1e Fig. NTA analysis showed that electroporated exosomes were slightly larger than non-electroporated exosomes and monodisperse (S1f Fig) consistent with findings by Tofoli et al. [24].

### Improved uptake of exosomal doxorubicin

**Fig 1:**
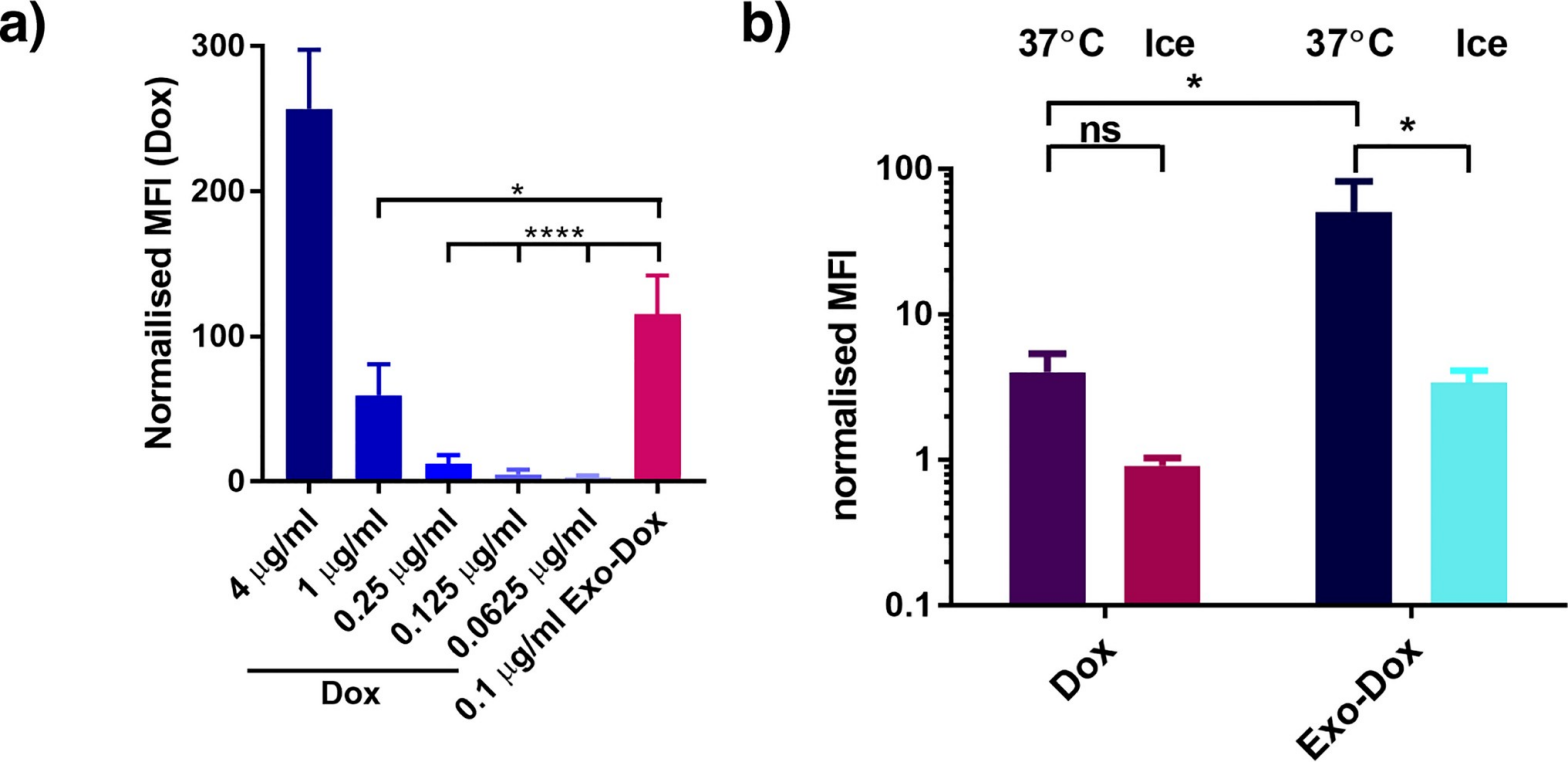
Improved uptake of exosomal doxorubicin. (a) HEK293 cells were incubated with different concentrations of free doxorubicin (Dox) or exosomal doxorubicin (Exo-Dox) in duplicate at 37 °C for 4 h. Uptake was analysed by flow cytometry, average mean fluorescence intensity (MFI) values were calculated and normalised to the MFI value obtained by incubating cells with an equivalent volume of PBS as control (b) HEK293 cells were incubated with free doxorubicin (Dox) or exosomal doxorubicin (Exo-Dox) for 4 h either at 37 °C or on ice in duplicate. Uptake was analysed by flow cytometry as described in a) n = 3; data is presented as mean +/-SD *p<0.05 and ****p<0.0001, ns non significant.

Free doxorubicin or exosomal doxorubicin (Exo-Dox) was incubated with HEK293 cells for 4 h at 37°C and the fluorescence signal, attributed to presence of Dox associated with the cells was detected using flow cytometry. These experiments revealed that approx. 15 times more free Dox is required to elicit an equivalent signal compared to Exo-Dox (Fig 1a). We next explored whether uptake of Dox was temperature dependent. When recipient HEK293 cells were incubated with Dox or Exo-Dox for 4 hours at 37°C or on ice, a significant decrease in recipient cell fluorescence was observed upon incubation on ice for cells treated with Exo-Dox. Moreover, the lower temperature had a greater impact on the uptake of Exo-Dox compared to free Dox (Fig 1b) which suggests a difference in the uptake mechanism. The increased rigidity of membranes at lower temperatures could explain the reduced uptake of free Dox entering cells by passive diffusion [30]. The significantly stronger reduction in uptake for Exo-Dox suggests the involvement of active transport in Exo-Dox uptake, in line with previous observations [15].

### Rapid kinetics of exosomal doxorubicin uptake *in vitro*, compared to other doxorubicin formulations

**Fig 2:**
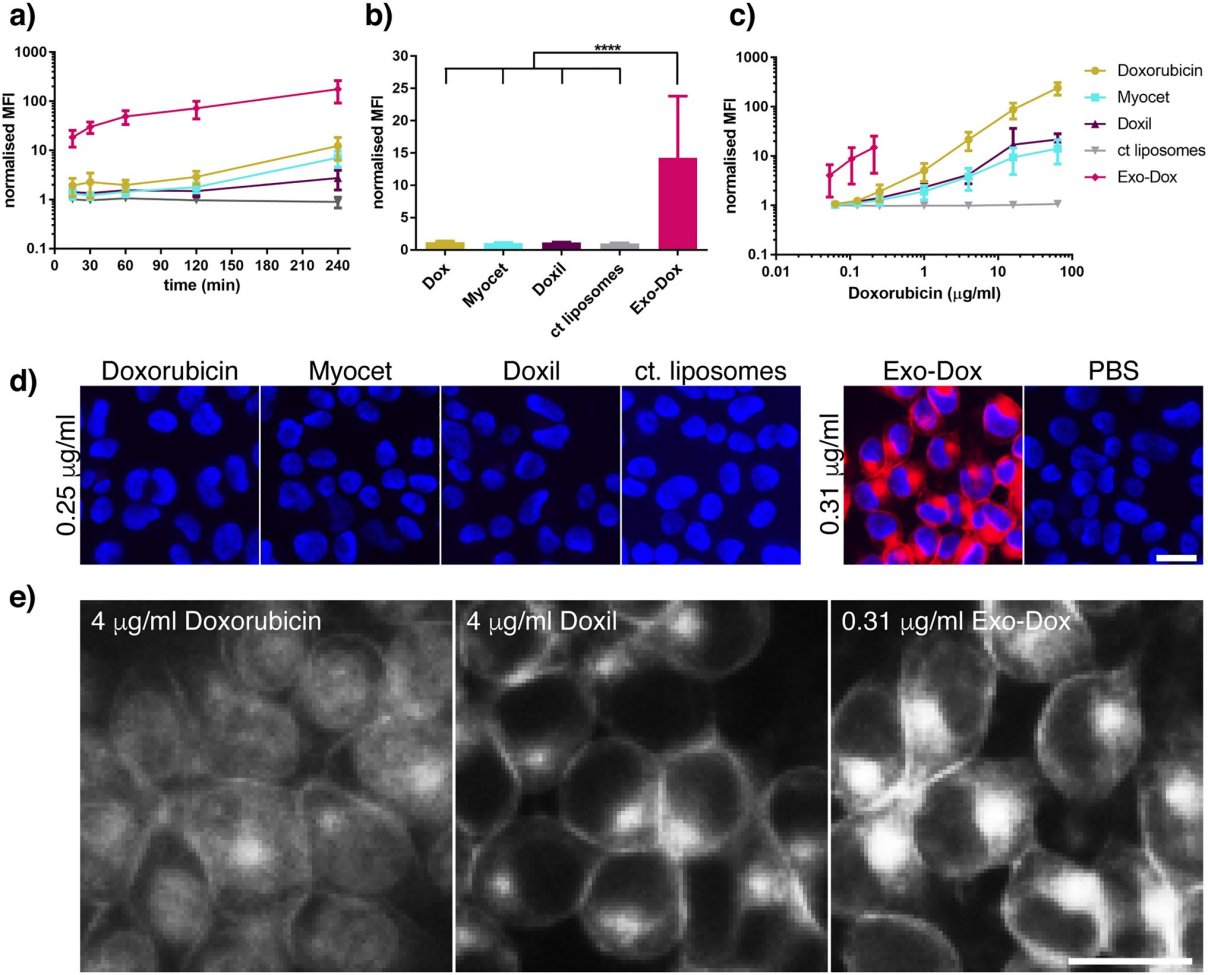
Rapid internalisation of exosomal doxorubicin. (a) HEK293 cells were incubated with 0.25 μg/ml Dox, Exo-Dox, liposomal formulations of Dox or PBS as control in duplicate for increasing amounts of time. Doxorubicin uptake was analysed by flow cytometry as described in Fig 1. (b) uptake was performed as described in (a) for 15 min. (c) HEK293 cells were incubated with increasing amounts of Dox, Exo-Dox, liposomal formulations of Dox or PBS for 15 min and uptake was assessed by flow cytometry analysis. (a,b,c) MFI values were normalised to MFI values obtained by incubation in PBS. All experiments were performed n = 3 times, data is presented as mean +/-SD, ****p<0.0001. (d) HEK293 cells were incubated with Dox, Exo-Dox, liposomal formulations of Dox (red) at concentrations indicated for 15 min followed by staining of the nuclei with Hoechst (blue). Uptake was analysed by epifluorescence microscopy; (e) experiment was performed as in (d), images for Dox concentrations that result in comparable recipient cell fluorescence are shown. (d,e) representative images from one (out of three) independent experiments are shown. Scale bar in microscopic images: 10 μm.

To analyse uptake of different formulations of doxorubicin further, a timecourse experiment was performed. Dox was compared to liposomal Dox (Myocet), Dox in pegylated liposomes (Doxil) and Exo-Dox at the same concentration. Over the 4 h incubation the cellular fluorescence for all Dox formulations increased; the uptake of Dox, Myocet and Exo-Dox increased 6–10 fold, whereas the uptake of Doxil only increased 3-fold (Fig 2a). Most strikingly, the uptake of Exo-Dox could be observed in the recipient cells after just 15 min incubation, the earliest timepoint tested, whereby all other formulations resulted in recipient cell fluorescence comparable to the PBS control at this timepoint (Fig 2a and 2b). Taken together, these results suggest a rapid and active internalisation mechanism for Exo-Dox. To exclude saturation of uptake pathways, a dose-response curve was generated using all Dox formulations. Fig 2c shows that recipient cell fluorescence can be observed after treatment with Dox or liposomal Dox (Myocet and Doxil) for 15 min, however, a 20- to 80-fold higher concentration of these formulations is required to elicit a similar signal to that seen for Exo-Dox. This titration also demonstrates that saturation of uptake pathways for these drugs were not limiting in our initial analysis of uptake at lower doxorubicin concentrations. Titration experiments show one limitation of our analysis due to the maximum achievable concentration of ~1.5 μg/ml of Dox in loaded exosomes.

We next assessed the intracellular distribution of doxorubicin by epifluorescence microscopy and found that free Dox had a diffuse distribution in the cytoplasm and nucleus whereas liposomal Dox and Exo-Dox showed an accumulation in discrete spots in addition to plasma membrane staining (Fig 2d and 2e). In line with the flow cytometry experiments, a lower concentration of Dox (~10-fold) was required to obtain a comparable staining intensity when incorporated into exosomes; Myocet uptake could only be visualised by microscopy at the highest concentration tested (S2 Fig).

Several active internalisation routes such as clathrin-dependent endocytosis, caveolae-based uptake or micropinocytosis have been observed for exosomes [15, 18, 31, 32] with preferential routes potentially defined by the cell type examined. To further explore the uptake as well as the nature of the large punctate structures that Exo-Dox was found localise to shortly after internalisation, Exo-Dox was co-incubated with fluorescent ligands routinely used to mark different active endocytic uptake routes and intracellular localisation was determined by confocal microscopy (S3 Fig). After 15 min the majority of Exo-Dox was found to be intracellular, as observed by delineating the plasma membrane using labelled Wheat-germ agglutinin (WGA). The most significant co-localisation, albeit partial, was found with CholeraToxin B subunit (CTxB) which enters cells via caveolae; in addition, a low level of co-localisation was found with fluorescently labelled transferrin (Tf), a marker used for clathrin-mediated endocytosis. These results suggest that the large intracellular structures seen in the epifluorescene microscopy are endosomes. Dextran enters cells through micropinocytosis; Dextran-filled endosomes did not co-localise with Exo-Dox filled endosomes indicating pathway selectivity in Exo-Dox uptake. Taken together, these results demonstrated that Exo-Dox is taken up into cells rapidly and with higher efficiency compared to other doxorubicin formulations; endocytosed Exo-Dox accumulates in endocytic structures immediately after uptake into HEK293 cells.

### Exosomal doxorubicin allows high intracellular accumulation of doxorubicin

**Fig 3:**
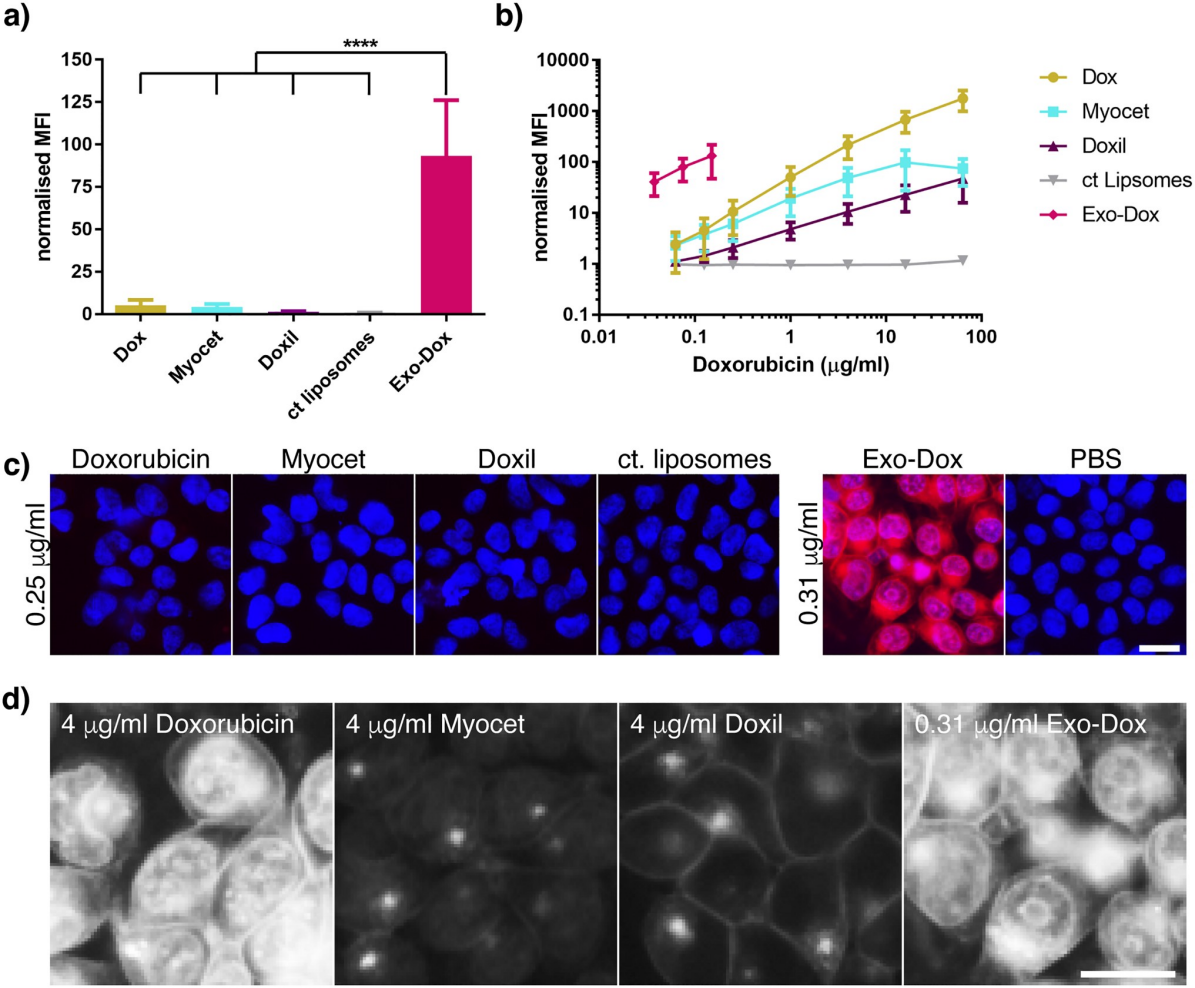
Sustained uptake of exosomal doxorubicin causes a high cumulative concentration. (a) HEK293 cells were incubated with 0.25 μg/ml Dox, liposomal Dox or Exo-Dox for 4 h at 37 °C or in duplicate. Cellular uptake was analysed as described in Fig 1. (b) HEK293 cells were incubated with increasing amounts of Dox, Exo-Dox, liposomal formulations of Dox or PBS and uptake was determined by flow cytometry analysis. (a,b) MFI values were normalised to MFI values obtained by incubation in PBS. Experiments were performed n = 3 times, data is presented as mean +/-SD, ****p<0.0001. (c) HEK293 cells were treated with Dox, Exo-Dox, liposomal formulations of Dox (red) at concentrations indicated for 4 h followed by Hoechst staining of the nuclei (blue). Uptake was analysed as described in Fig 2. (d) Uptake experiment was performed as in (c), images for Dox concentrations that result in comparable recipient cell fluorescence are shown. Representative images from (one out of three) independent experiments are shown. Scale bar: 10 μm.

We then investigated the intracellular accumulation of Exo-Dox and other Dox formulations, after a longer 4 h incubation using the flow cytometry assay. Again, the fluorescent signal from Exo-Dox was significantly higher than with the other drug formulations indicating that the effect observed at 15 min could be sustained over a few hours. In addition, the fold-change compared to other formulations was much higher compared to the 15 min timepoint (18-fold compared to free Dox, 21-fold and 65-fold compared to Myocet and Doxil, respectively) (Fig 3a). A dose-response curve showed that uptake pathways were not saturated in the presence of lower concentrations of the doxorubicin formulations used in the assay (Fig 3b). Of note, both liposomal formulations were taken up less efficiently and accumulated less efficiently in the recipient cells compared to Exo-Dox or the free drug. We next explored the intracellular fate of Dox administered in various formulations for 4 h. Surprisingly, microscopic analysis of treated cells showed a diffuse cytoplasmic and nuclear pattern for both Dox and Exo-Dox, whereas Myocet and Doxil mostly retained the punctate distribution that was observed at an earlier timepoint (Fig 3c and 3d). This demonstrates that after initial uptake Exo-Dox can be rapidly released from endosomes.

### Time-lapse videomicrosopy shows rapid onset of mitochondrial enlargement and apoptosis following exosomal doxorubicin treatment

**Fig 4:**
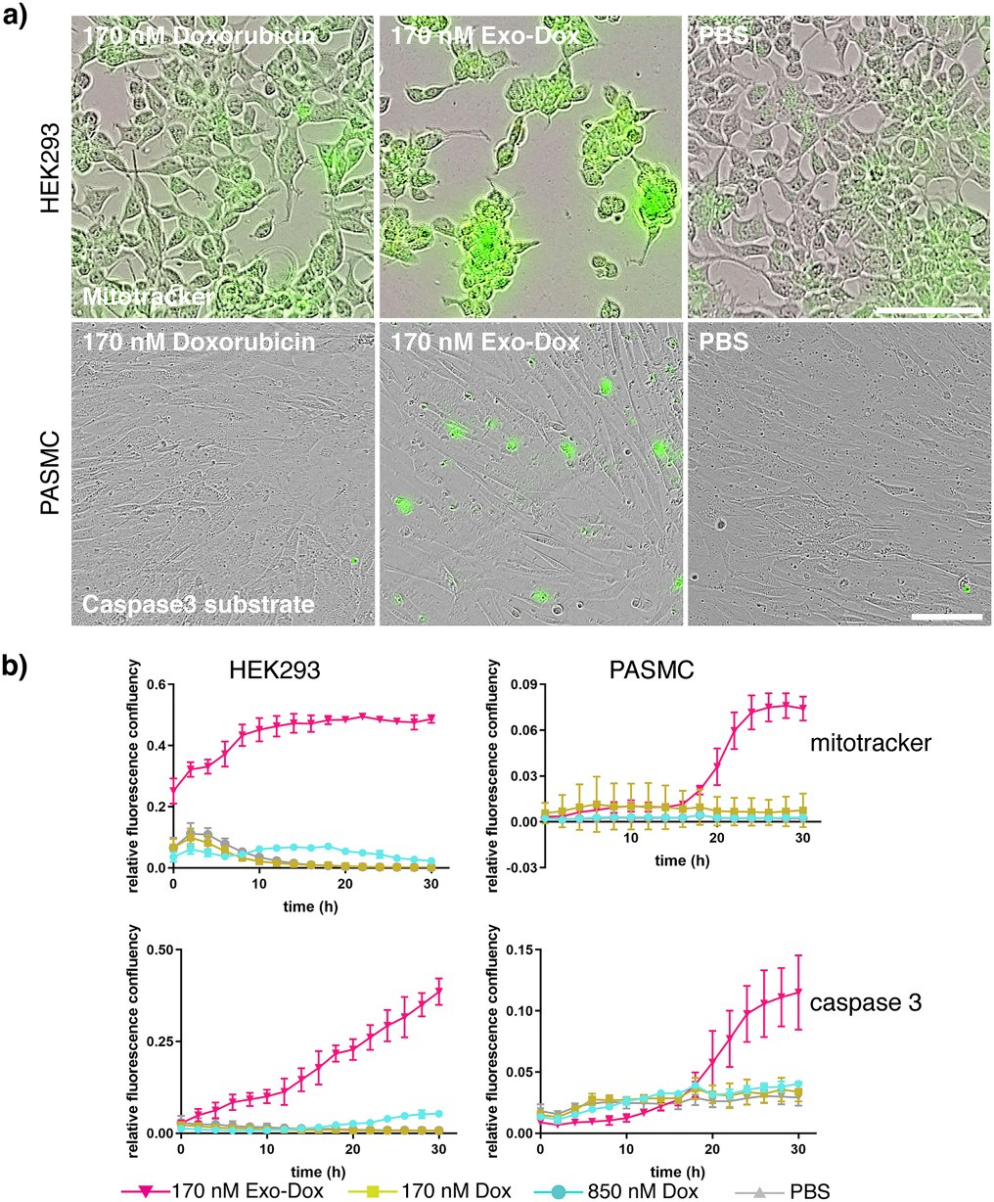
Time-lapse videomicroscopy shows that Exo-Dox causes enhanced mitochondrial swelling and apoptosis. HEK293 and PASMC were treated with 170 nM Exo-Dox or Dox at the same and a 5-fold higher concentration (850 nM) in triplicate. Mitochondrial morphology was analysed by addition of mitotracker and apoptotic cell death was analysed by addition of caspase 3 sensitive substrate DEVD-nucview488 using time-lapse videomicroscopy for which cells were imaged every 2 h over the course of 30 h. Fig 4a shows representative images taken at the 24 h timepoint of HEK293 (top panel, mitotracker staining) or PASMC cells (bottom panel, caspase 3 substrate staining). Relative fluorescence intensity was determined by divinding fluorescence confluency by phase confluency. Fig 4b shows a timecourse of relative fluorescence intensity for mitochondrial dye mitotracker and caspase 3 activity. One (out of three) representative experiments is shown. Data is displayed as mean +/-SEM; scale bar: 100 μm.

**Fig 5:**
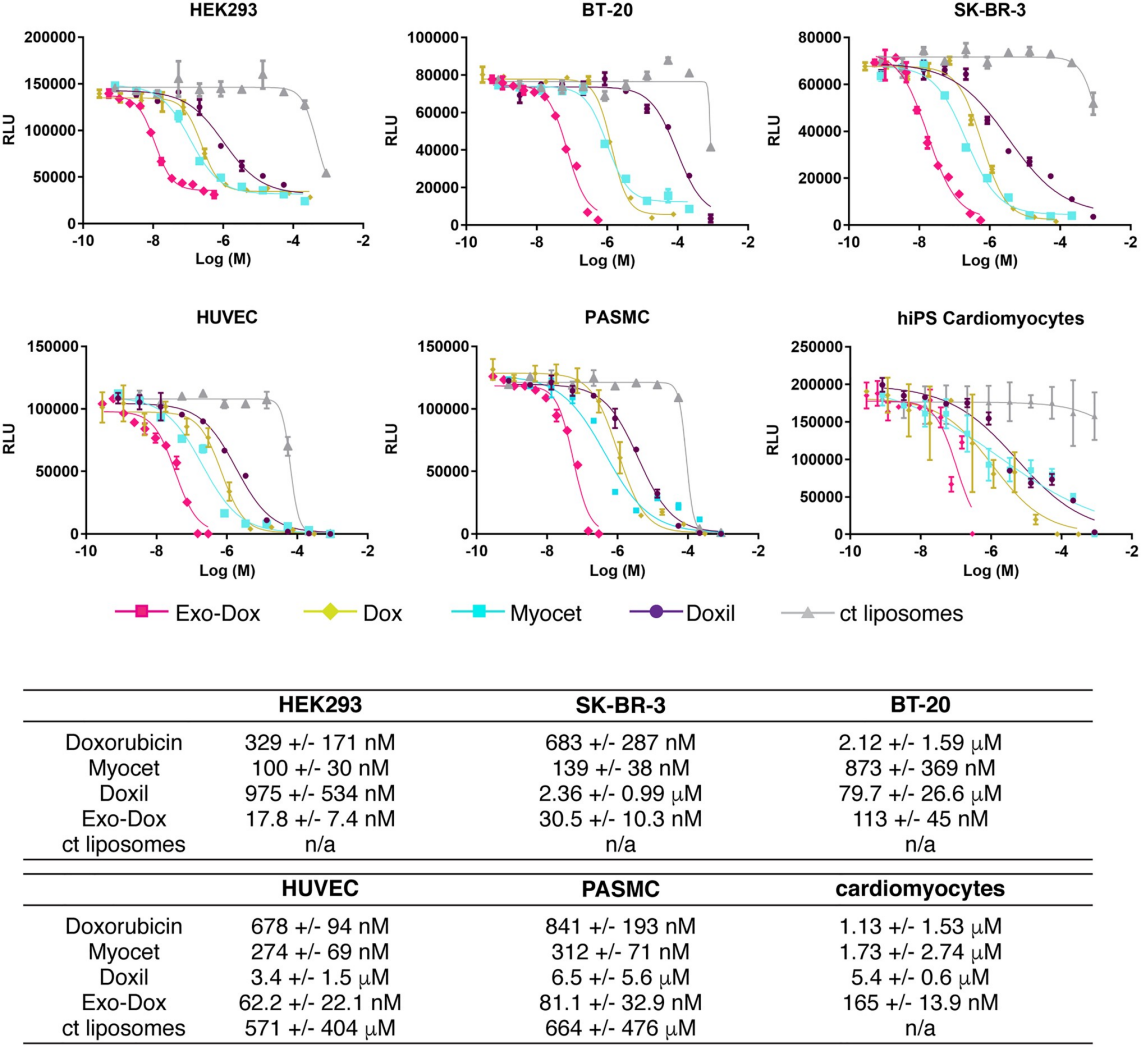
Exosomal doxorubicin is more potent than free doxorubicin or liposomal formulations of doxorubicin in a wide range of cultured cells. HEK293, SK-BR-3, BT-20, HUVEC, PASMC and hiPS cardiomyocytes were treated with increasing amounts of Dox, Exo-Dox, Myocet, Doxil or control liposomes in duplicate for 48 h (HEK293, SK-BR-3, BT20) or 72 h (HUVEC, PASMC, iCell cardiomyocytes). Cellular ATP content as measure of cell viability was determined using luminescent cell titre Glo assay. Results were plotted in GraphPad Prism 6 using a 4-parameter logistic equation to give IC50 values. Representative graphs are shown in (a). (b) table summarising mean +/- SEM. IC50 values from n = 3 independent experiments performed in HEK293, SK-BR-3, BT-20, HUVEC and PASMC and n = 2 independent experiments performed in hiPS cardiomyocytes.

A hallmark of Dox treatment is mitochondrial enlargement and dysfunction causing a loss of cellular ATP and build-up of free radicals which are triggers for apoptotic cell death mediated by activation of executioner caspase-3 [33–35]. We thus wanted to visualise the induction of these phenotypes by Exo-Dox using specific probes; Mitotracker to stain mitochondria independent of the membrane potential and the cleavage of the fluorogenic substrate DEVD-Nucview488 by activated caspase-3 [36]. In these experiments we incubated Dox with HEK293 cells, as well as low passage human PASMC cells which have a high mitochondrial content, and monitored their phenotypes using time-lapse video-microscopy (Fig 4). Free Dox was added to cells at a concentration (170 nM) lower than the IC_50_ for both cell types (see Fig 5) alongside the equivalent concentrations of Exo-Dox. Our experiments clearly showed that in both cell lines mitochondrial swelling occurred within 24 h of treatment and was more pronounced in Exo-Dox treated compared to Dox treated cells (Fig 4). Whilst swelling of mitochondria could be observed almost immediately after treatment in HEK293 cells, this phenotype was delayed in PASMC cells. This could be explained by a lower sensitivity of PASMC cells to Dox (Fig 5), compared to HEK293 cells but also by a lower uptake efficiency of Dox by PASMC cells, as measured by flow cytometry (compare S5b Fig to Fig 3a). As potential consequence of mitochondrial dysfunction, the onset of apoptotic cell death driven by caspase activity occurred concomitantly with mitochondrial swelling in both cell lines tested and was more pronounced in Exo-Dox treated cells. Of note, similar phenotypes could not be observed in either cell line when incubated with five times the amount of free Dox. The established apoptosis-inducer Camptothecin was used as a positive control. The fraction of cells showing caspase activity was similar in Exo-Dox and Camptothecin-treated cells (compare Fig 4a and S4a fig).

### Improved potency of exosomal doxorubicin in cultured immortalised and primary cell lines

Our time-lapse video microscopy data showed that Exo-Dox causes a greater level of mitochondrial swelling and results in apoptosis in a greater fraction of cells compared to free Dox, but the method is limited in the number of conditions that can be tested. To compare potency of Exo-Dox to other formulations, we employed a standard cell viability assay measuring cellular ATP content. A small panel of immortalised cell lines (HEK293, SK-BR-3 and BT-20) were treated with Exo-Dox, free Dox or liposomal Dox for 48 h (Fig 5). The IC_50_ values obtained from 3 independent experiments summarized in Fig 5 show that Exo-Dox is significantly more potent than other formulations of Dox (~20-fold compared to Dox, ~5-fold compared to Myocet and >50-fold compared to Doxil). As cardiotoxicity is the major side effect of doxorubicin treatment, we also examined hiPS cardiomyocytes that were obtained by differentiating human iPS cells, as well as low passage human cells HUVEC or PASMC which were treated in an identical manner and analysed by cell viability assay after 72 h (Fig 5). Overall, similar data to the cultured cell lines were obtained; Exo-Dox is more potent compared to Myocet and free Dox, Doxil is the least potent formulation demonstrating that the superior efficacy of Exo-Dox can be achieved in slow or non-dividing cells that have not been immortalised or are of tumour origin. Uptake of Exo-Dox was visualised by flow cytometry and microscopy for PASMC (S5b Fig) or hiPS cardiomyocytes (S6a Fig). These experiments demonstrate the improved uptake of Exo-Dox compared to other Dox formulations, which correlates well with the overall improvement of Exo-Dox potency compared to that seen for free drug in primary cells (7-10-fold lower) and immortalised cell lines (~20-fold lower).

## Discusssion

Several studies have explored the utility of exosomes for the delivery of small molecule drugs both *in vitro* and *in vivo* [19, 22, 37]. Indeed, these studies have demonstrated that exosomes loaded with small molecule therapeutics possess superior therapeutic effects *in vitro* compared to the free drug or encapsulated carriers such as liposomes [21, 23]. Few studies, however, have investigated the reason for this superior potency which is a key to the further development of drug-loaded exosomes for use in the clinic. In this study we chose to focus on doxorubicin as a cargo, as its uptake into cells can be readily monitored by flow cytometry and microscopy by virtue of its intrinsic fluorescence. We compared the uptake of free Dox to encapsulated Dox formulations, and demonstrated that Exo-Dox has superior uptake kinetics. Furthermore, it is the only formulation that can be detected in cells after just 15 minutes of exposure at low drug concentrations and this increase in uptake is sustained over several hours. Notably, following cellular uptake, Exo-Dox is released from the endocytic compartment within a few hours of treatment and after release is able to efficiently induce cellular events typically associated with Dox administration, such as mitochondrial enlagement and onset of apoptosis, at much lower concentrations compared to the free drug. When IC50 values were determined using a standard cell viability assay, Exo-Dox was 7–20 fold more potent compared to the free drug and 4–10 times more potent compared to non-pegylated liposomal Dox (Myocet).

To encapsulate Dox into exosomes, we optimised an electroporation-based method which, although successful, could be considered a harsh process that could compromise exosomal membrane integrity and therefore the ability to interact with and enter cells. We were able to show, however, that Exo-Dox is monodisperse in NTA analysis and is capable of undergoing rapid active uptake along pathways that have previously been established as internalisation routes for exosomes (Fig 2 and S1 and S2 Figs [15, 18, 31, 32]). Co-incubation experiments with endocytic tracers (S3 Fig) suggest caveolae as well as receptor-mediated endocytosis as entry pathways for Exo-Dox into HEK293 cells. Our data aligns well with Yang et al. [38] determining both uptake pathways to be implicated in the uptake of electroporated exosomes into MCF-7 cells.

More interestingly, Exo-Dox uptake is much faster and more efficient compared to liposomal formulations. The slow cellular uptake of pegylated liposomal doxorubicin (Doxil) can be explained by the surface presence of PEG, a modification that was specifically introduced to prevent unspecific cellular uptake, to allow longer circulation times and preferential uptake into tumour cells due to the EPR effect [39]. Also non-pegylated liposomal doxorubicin (Myocet) uptake is less efficient than the free drug which is most likely due to the limited interactions that occur between the integral lipids of the liposome and the cell membrane. As exosomes are derived from natural cellular membranes, they possess cellular adhesion proteins such as integrins as well as proteoglycans and lectins which not only promote initial adhesion to recipient cells, but most likely contribute to rapid initiation of cellular uptake [40]. Indeed, experiments performed by Longatti et al., Smyth et al. and Koojimans et al. comparing the uptake of naïve and proteinase K-treated exosomes show a significant reduction of cellular uptake in the absence of exosomal surface proteins [25, 41, 42].

Another intriguing finding was the rapid re-distribution of Dox from endosomes to the cytoplasm (Fig 3c and 3d). This rapid release was previously reported by Kim et al. for Paclitaxel-loaded exosomes where the authors suggest that a fraction of Paclitaxel is peripherally associated with exosomes and offers a readily releasable pool [23]. In addition, natural membranes might be able to release the drug faster in the low pH environment of the endosome compared to artificial liposomes that commonly are loaded using pH gradients [43, 44] and in their composition are designed to retain the drug for prolonged periods of time until it could be deposited in tumours penetrating the leaky tumour vasculature (EPR effect).

Our findings further demonstrate that Exo-Dox can maintain favourable high intracellular levels over a prolonged incubation time (Fig 3). Kim et al. previously showed that Exo-Dox is not subject to efflux via multidrug resistance (MDR) transporters in the same way as free Dox, serving as an explanation for the elevated cellular levels after 4 h of incubation [23]. Despite its initial poor uptake and slow endosomal release (Figs 2 and 3) we found Myocet was more potent compared to free Dox and Doxil (Fig 5) in a 48 h test, aligning well with published data [45] on better release of Dox from non-pegylated compared to pegylated liposomes and further supporting the notion that liposomal Dox is also protected from MDR mediated export [46]. The high intracellular Dox levels achieved by Exo-Dox likely account for the increased cellular toxicity of Exo-Dox causing mitochondrial swelling and apoptosis (Fig 4) as well as an overall increased potency in cell viability assays (Fig 5).

Overall, IC50 values obtained in this study match published data for Dox [47] and the reported 10-20-fold difference in potency between free and exosome encapsuled drug has been reported in a study using Exo-Pax; Kim et al [21] determined a similar 10–20 fold improved potency upon Paclitaxel incorporation into exosomes suggesting that the potency improvement can be achieved for other small molecule drugs. In more detail, our results show a higher potency in cultured cells (~20-fold) lines compared to human primary cells at low passage (7-10-fold) (Fig 5). In addition to a lower uptake in these cells (S5 and S6 Figs), this might be related to a second major mechanism of cellular toxcicty of Dox, namely its intercalation into DNA and binding to DNA associated enzymes such as topoisomerase thus blocking DNA replication [48]. All cultured cell lines used in this study are fast dividing and have an aberrant p53 pathway which most likely enhances the toxic effects of Dox on DNA replication in these cell lines [49]. It can be assumed that human cells at passage 2 or 3 have an intact p53 pathway.

The therapeutic window of a drug is defined as a drug concentration range that provides efficacy without causing unacceptable toxicity; a large therapeutic window is thus advantageous for safe use of a drug in the clinic [50]. In our experiments we demonstrated a 7-20-fold increase in potency of Exo-Dox compared to free drug or liposomal formulations, indicating that therapeutic effects could be achieved at a much lower dose. We also observed this increased potency in hiPS cardiomyocytes suggesting that that the therapeutic window of Exo-Dox might not necessarily be greater compared to conventional formulations. This, however, assumes that target and cardiac cells will receive the same dose of drug-loaded exosomes, which may not necessarily the case. Indeed, although Tofoli et al.[24] observed a comparable potency of Exo-Dox and free Dox in cultured MDA-MB-231 and HCT116 cells, Exo-Dox led to very little cardiotoxicity in a mouse study. In a follow up study, the same authors find that Exo-Dox, unlike free Dox, is very inefficient in passing a reconstituted myocardial endothelial monolayer; thus due to the tight junction structure in the heart’s blood vessels, Exo-Dox uptake is most likely shifted away from deposition in the heart [37]. This altered biodistribution could result in higher tumour vs heart accumulation as exosomes are taken up into tumours using the EPR effect in the leaky tumour vasculature. Additional studies using exosomes from different sources suggest that the heart is not a preferred target organ for exosome uptake in mouse models [22, 51, 52]. Indeed, the same studies show that *in vivo*, exosomes isolated from different cell types show a differential tissue distribution partly dependent on the cellular source. This natural tropism could be exploited to generate a toolbox of therapeutic exosomes targeting different tissues.

To permit systemic administration of exosomally encapsulated drugs, and enable selective uptake into the desired cell type or tissue, targeting ligands that bind to cell surface receptors can be incorporated, which has been demonstrated for Her2-targeted Dox-liposomes [53]. Whilst exosomal targeting has not been explored to a great extent, a limited number of naturally occurring ligand-receptor interactions have so far been shown to improve uptake of exosomes into cells expressing cognate receptors (reviewed in Gyorgy et al. [54]). For example, Alvarez-Erviti et al have successfully demonstrated that exosomes modified to contain RVG peptide could target exosomes to the brain resulting in productive uptake into neuronal cells expressing nACh [19]. More promising however is the addition of targeting ligands that can be engineered to bind to any cognate receptor of choice, such as nanobodies or single chain variable fragments (scFv). These ligands can be incorporated into the exosomes by genetic manipulation of the exosome-producing cell line or post-exosome production using conjugation technologies such as click chemistry [25, 41, 55]. In studies using exosomes displaying anti-Her2 scFv, improved uptake into cells expressing high levels of Her2 could be demonstrated *in vitro* [25] and *in vivo* (our unpublished data).

Local application is an alternative strategy for the delivery of small molecule-loaded exosomes to enable selective uptake. Studies with curcumin-loaded exosomes applied intranasally show that loaded exosomes do not only penetrate into the brain but also prevented LPS-induced brain inflammation in mice [20]. The rapid uptake into cells could prevent or reduce its transport into other tissues where it could cause side effects.

In summary, we have conducted a comprehensive analysis of the uptake of Dox into cells, and the subsequent impact on cell phenotype and viability. We have provided compelling evidence that Exo-Dox is rapidly and efficiently taken up into cells where it accumulates to high levels and demonstrates superior potency compared to the free drug or its liposomal formulations *in vitro*. Combined with their natural tropism, and the potential to engineer them for improved selectivity, small molecule loaded exosomes provide a novel means to efficiently deliver potent therapeutics with an improved therapeutic index, thereby improving patient outcomes.

## Supporting information

S1 Fig Characterisation of exosomes. (a) Comparison of viability of HEK293 cells cultured in serum-free Freestyle medium or MEM supplemented with 10% FBS over the course of 3 days (n = 2). (b) 10 μg total protein total cell extract or exosomes obtained by either filtration concentration or ultracentrifugation (UC) were analysed by immunoblotting using antibodies to exosome enriched proteins CD63, Alix or Tsg101 or contaminant markers calnexin and fibronectin, actin was utilised as ubiquitous marker. (c) The ratio of particle concentration (in e12 particles/ml) to total protein content (in mg/ml; determined by BCA assay) as a measure of purity were calculated. Analysis of n = 4 independent experiments visualised by box and whisker plot shows a 2-fold increase in purity of UC exosomes compared to exosomes isolated by filtration. Additionally, purity of UC preparations was slightly more reproducible. (d) representative NTA analysis of size distribution of exosomes isolated by filtration (left) or UC (right). (e) Dox loading efficiency expressed in μg Dox/particle n = 8. (f) representative NTA analysis of electroporated exosomes.
(TIFF)

S2 Fig Uptake of doxorubicin into HEK293 cells. HEK293 cells were incubated with Dox, Exo-Dox, liposomal formulations of Dox (red) at concentrations indicated for 15 min followed by staining of the nuclei with Hoechst (blue). Uptake was analysed by epifluorescence microscopy; representative images from one (out of three) independent experiments are shown; scale bar: 10 μm. (b).
(TIFF)

S3 Fig Co-incubation of Exo-Dox with endocytic tracers. HEK293 cells were co-incubated with 0.26 mg/ml Exo-Dox and 2.5 mg/ml Wheat-Germ-Agglutinin (WGA)-Alexa647 or 50 mg/ml Transferrin-Alexa647 or 1.25 mg/ml Choleratoxin B subunit (CTxB)-Alexa647 or 200 mg/ml Dextran-Alexa647 for 10 min at 37 °C, Hoechst33342 nuclear stain was added at 1 mg/ml and uptake was continued for 5 more min. Cells were washed twice in PBS and switched to culture medium containing FBS for immediate imaging on an Opera confocal imaging system using the same exposure settings for all treatments. Due to the differential uptake of the endocytic tracers, brightness and contrast were individually adjusted to give best images. Scale bar: 10 μm.
(TIFF)

S4 Fig Prolonged incubation of HEK293 cells with doxorubicin. HEK293 cells were treated with Dox, Exo-Dox, liposomal formulations of Dox (red) at concentrations indicated for 4 h followed by Hoechst staining of the nuclei (blue). Uptake was analysed as described in Fig 2d. Representative images from one (out of three) experiments are shown; scale bar: 10 μm.
(TIFF)

S5 Fig Uptake of Dox into PASMC and apoptosis control. (a) Apoptosis inducer Camptothecin was added to PASMC cells for 24h at concentrations as indicated in the figure in presence of a caspase 3 sensitive fluorogenic substrate, scale bar 100 μm, n = 1. (b) PASMC cells were treated with Dox, Exo-Dox, liposomal formulations of Dox at 0.25 μg/ml for 4 h; uptake was analysed by flow cytometry as described in Fig 1. n = 3, data is displayed as mean +/- SD, ****p<0.0001.
(TIFF)

S6 Fig Endocytosis of Exo-Dox into hiPS cardiomyocytes. (a) HEK293, BT-20 and SK-BR-3 cells were treated with increasing amounts of free Dox, Exo-Dox or an equivalent particle number of non-loaded control exosomes. Cellular ATP content as measure od viability was determined as in Fig 5; n = 1 data is presented as mean +/- SD. (b) hiPS cardiomyocytes were treated with Dox, Exo-Dox, liposomal formulations of Dox at 0.155 μg/ml for 4 h; uptake was analysed by flow cytometry as described in Fig 1. n = 3, data is displayed as mean +/- SD, ****p<0.0001. (c). hiPS cardiomyocytes cells were incubated with Dox, Exo-Dox, liposomal formulations of Dox (red) at concentrations indicated for 4 h followed by staining of the nuclei with Hoechst (blue). Uptake was analysed by epifluorescence microscopy; representative images from one (out of three) independent experiments are shown. (d) magnified images showing similar red fluorescence intensities from the panel in (c); scale bars: 10 μm.
(TIFF)

S1 Methods (DOCX)
