# Supplementary figures and images for: Exosomal delivery of doxorubicin enables rapid cell entry and enhanced in vitro potency
Source: PLoS One. 2019 Mar 29;14(3):e0214545. doi: 10.1371/journal.pone.0214545 (PMC6440694; doi:10.1371/journal.pone.0214545)

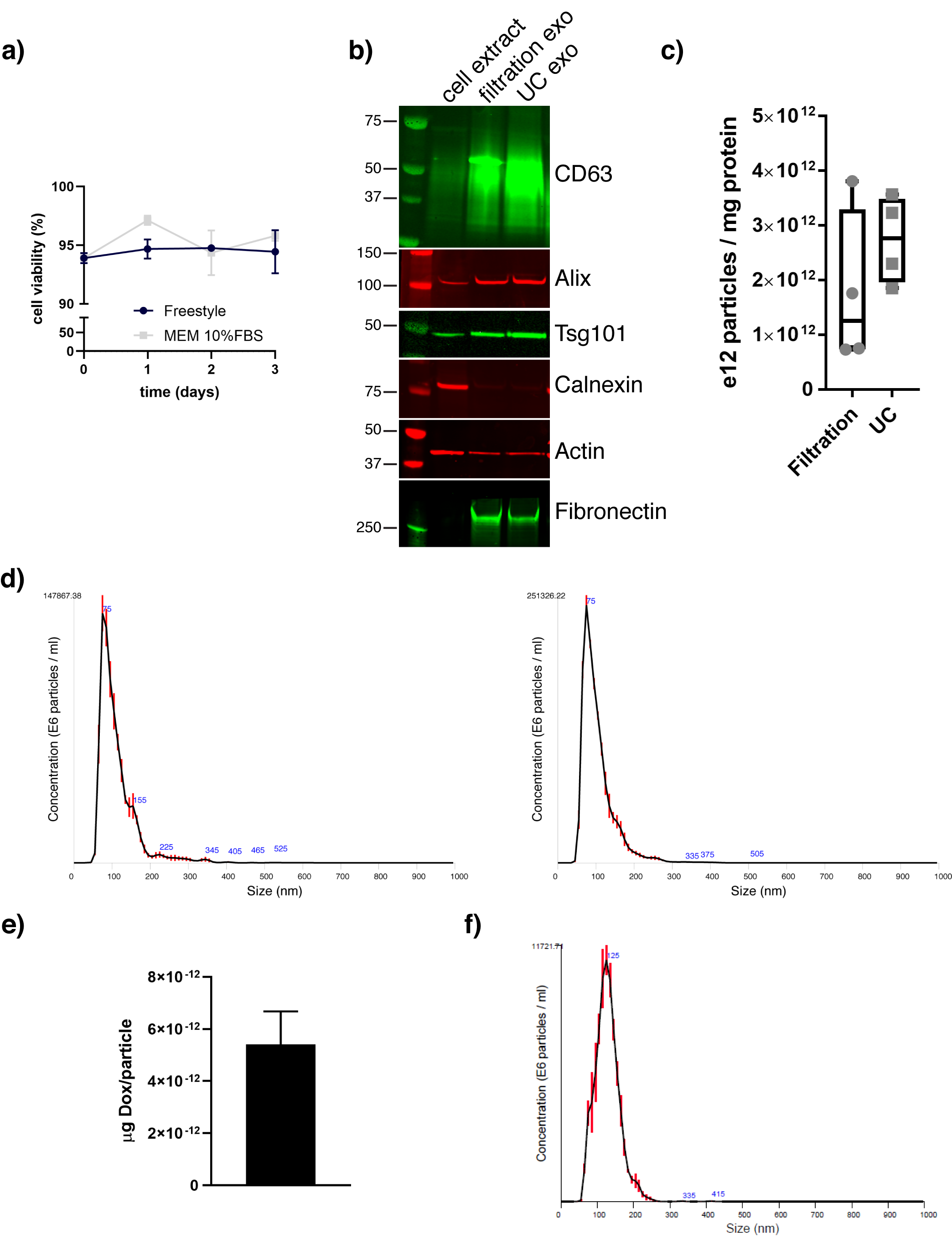

Supplement: S1 Fig — (a) Comparison of viability of HEK293 cells cultured in serum-free Freestyle medium or MEM supplemented with 10% FBS over the course of 3 days (n = 2). (b) 10 μg total protein total cell extract or exosomes obtained by either filtration concentration or ultracentrifugation (UC) were analysed by immunoblotting using antibodies to exosome enriched proteins CD63, Alix or Tsg101 or contaminant markers calnexin and fibronectin, actin was utilised as ubiquitous marker. (c) The ratio of particle concentration (in e12 particles/ml) to total protein content (in mg/ml; determined by BCA assay) as a measure of purity were calculated. Analysis of n = 4 independent experiments visualised by box and whisker plot shows a 2-fold increase in purity of UC exosomes compared to exosomes isolated by filtration. Additionally, purity of UC preparations was slightly more reproducible. (d) representative NTA analysis of size distribution of exosomes isolated by filtration (left) or UC (right). (e) Dox loading efficiency expressed in μg Dox/particle n = 8. (f) representative NTA analysis of electroporated exosomes. (TIFF) [file pone.0214545.s001.tiff]

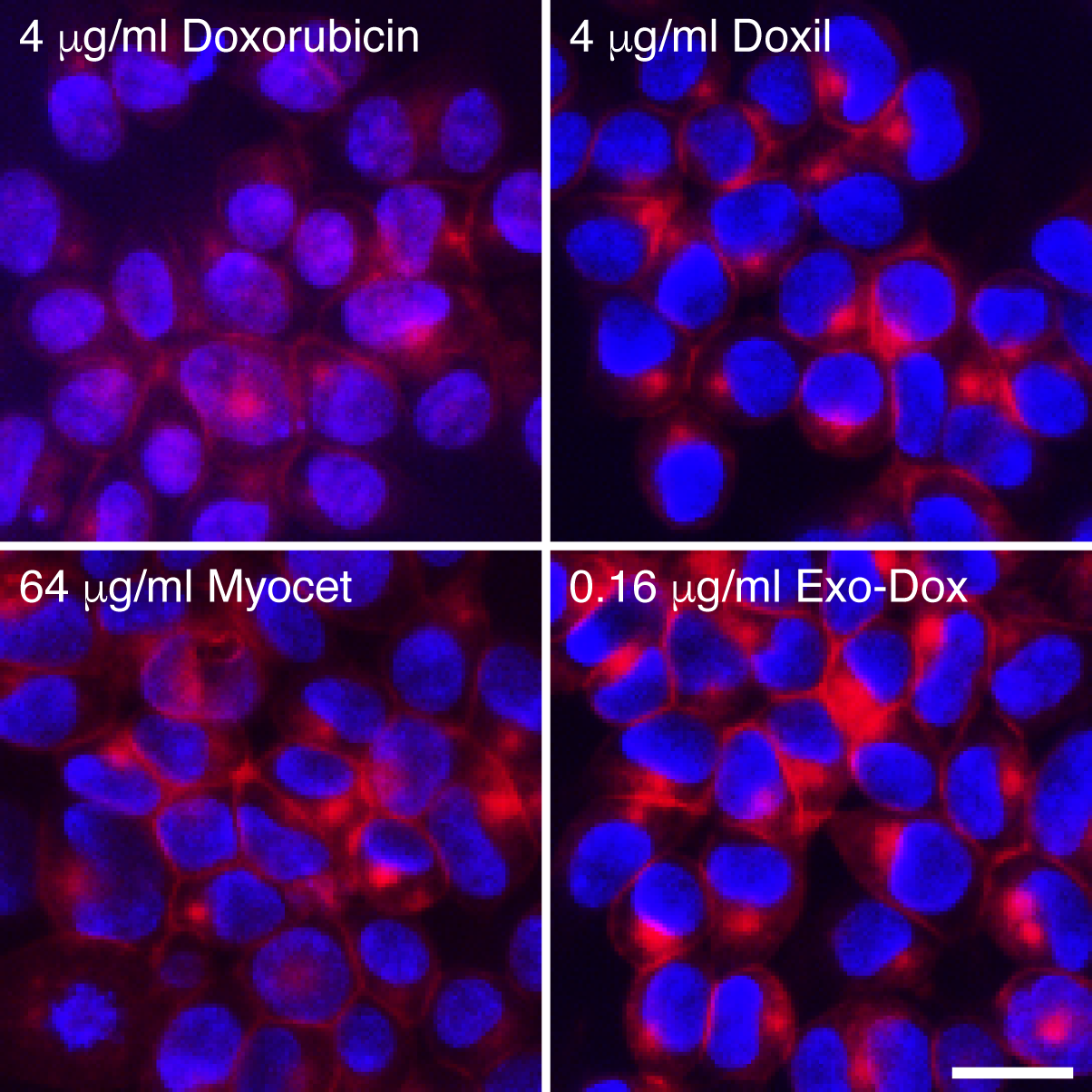

Supplement: S2 Fig — HEK293 cells were incubated with Dox, Exo-Dox, liposomal formulations of Dox (red) at concentrations indicated for 15 min followed by staining of the nuclei with Hoechst (blue). Uptake was analysed by epifluorescence microscopy; representative images from one (out of three) independent experiments are shown; scale bar: 10 μm. (b). (TIFF) [file pone.0214545.s002.tiff]

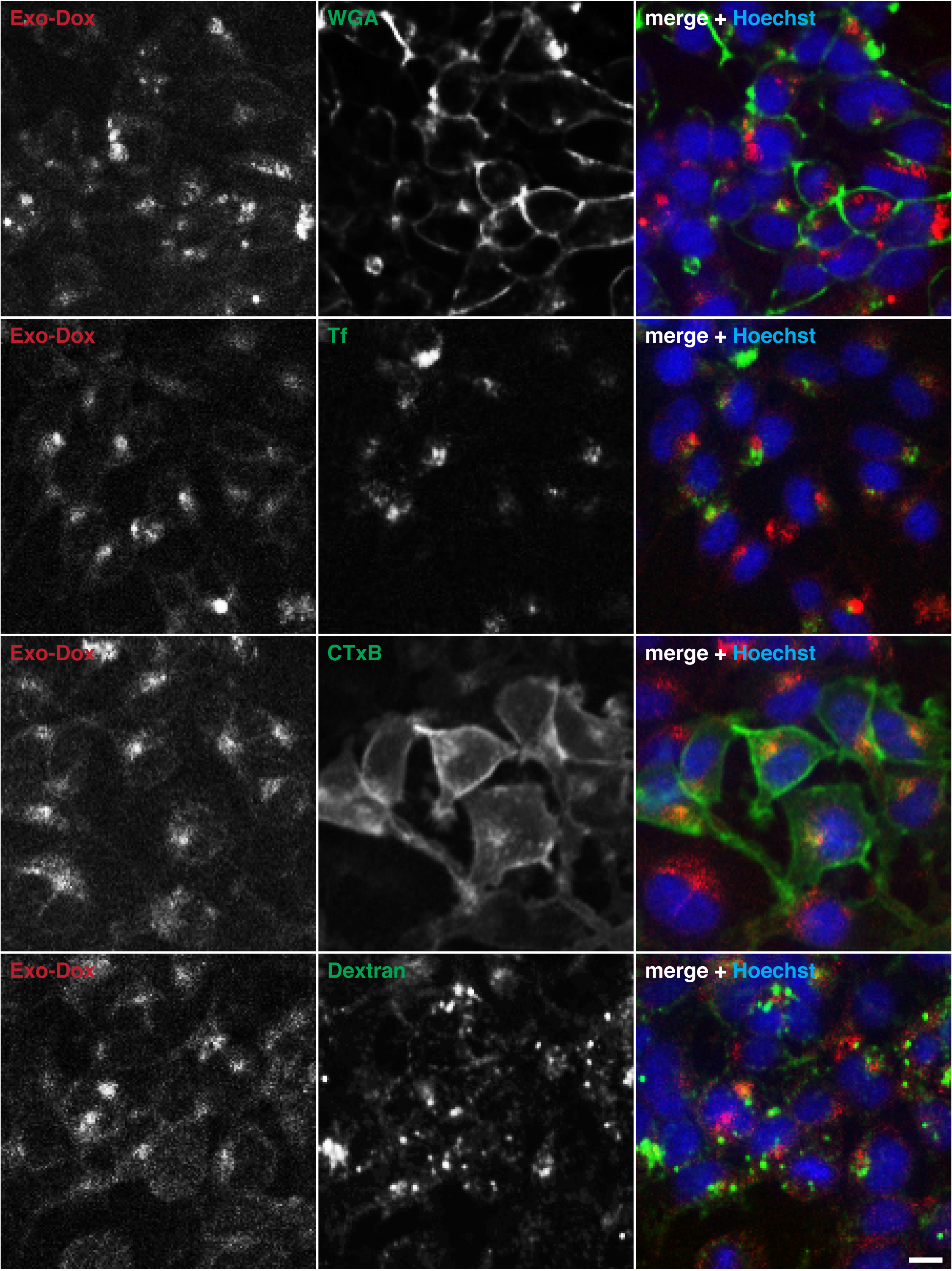

Supplement: S3 Fig — HEK293 cells were co-incubated with 0.26 mg/ml Exo-Dox and 2.5 mg/ml Wheat-Germ-Agglutinin (WGA)-Alexa647 or 50 mg/ml Transferrin-Alexa647 or 1.25 mg/ml Choleratoxin B subunit (CTxB)-Alexa647 or 200 mg/ml Dextran-Alexa647 for 10 min at 37 °C, Hoechst33342 nuclear stain was added at 1 mg/ml and uptake was continued for 5 more min. Cells were washed twice in PBS and switched to culture medium containing FBS for immediate imaging on an Opera confocal imaging system using the same exposure settings for all treatments. Due to the differential uptake of the endocytic tracers, brightness and contrast were individually adjusted to give best images. Scale bar: 10 μm. (TIFF) [file pone.0214545.s003.tiff]

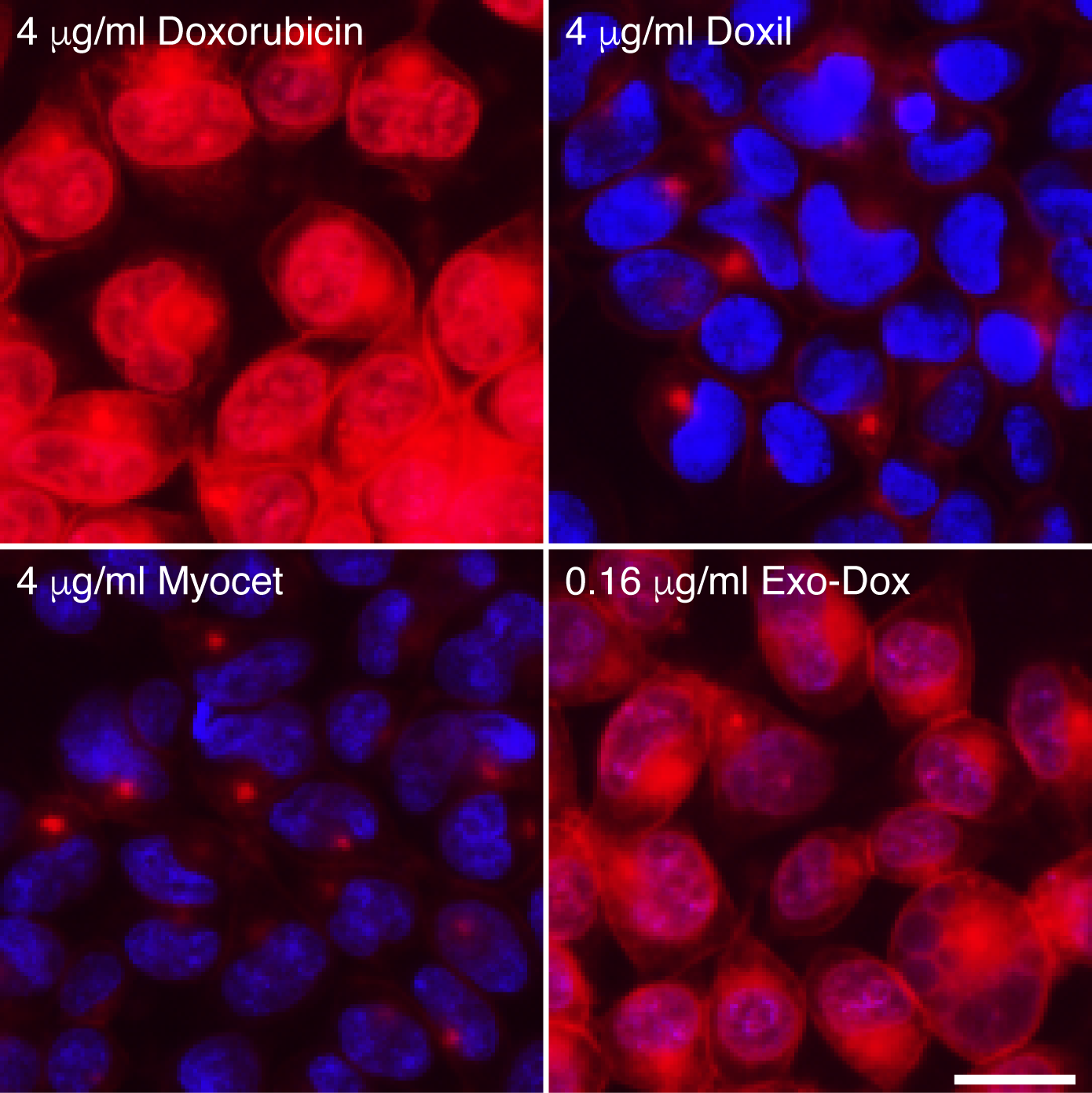

Supplement: S4 Fig — HEK293 cells were treated with Dox, Exo-Dox, liposomal formulations of Dox (red) at concentrations indicated for 4 h followed by Hoechst staining of the nuclei (blue). Uptake was analysed as described in Fig 2d. Representative images from one (out of three) experiments are shown; scale bar: 10 μm. (TIFF) [file pone.0214545.s004.tiff]

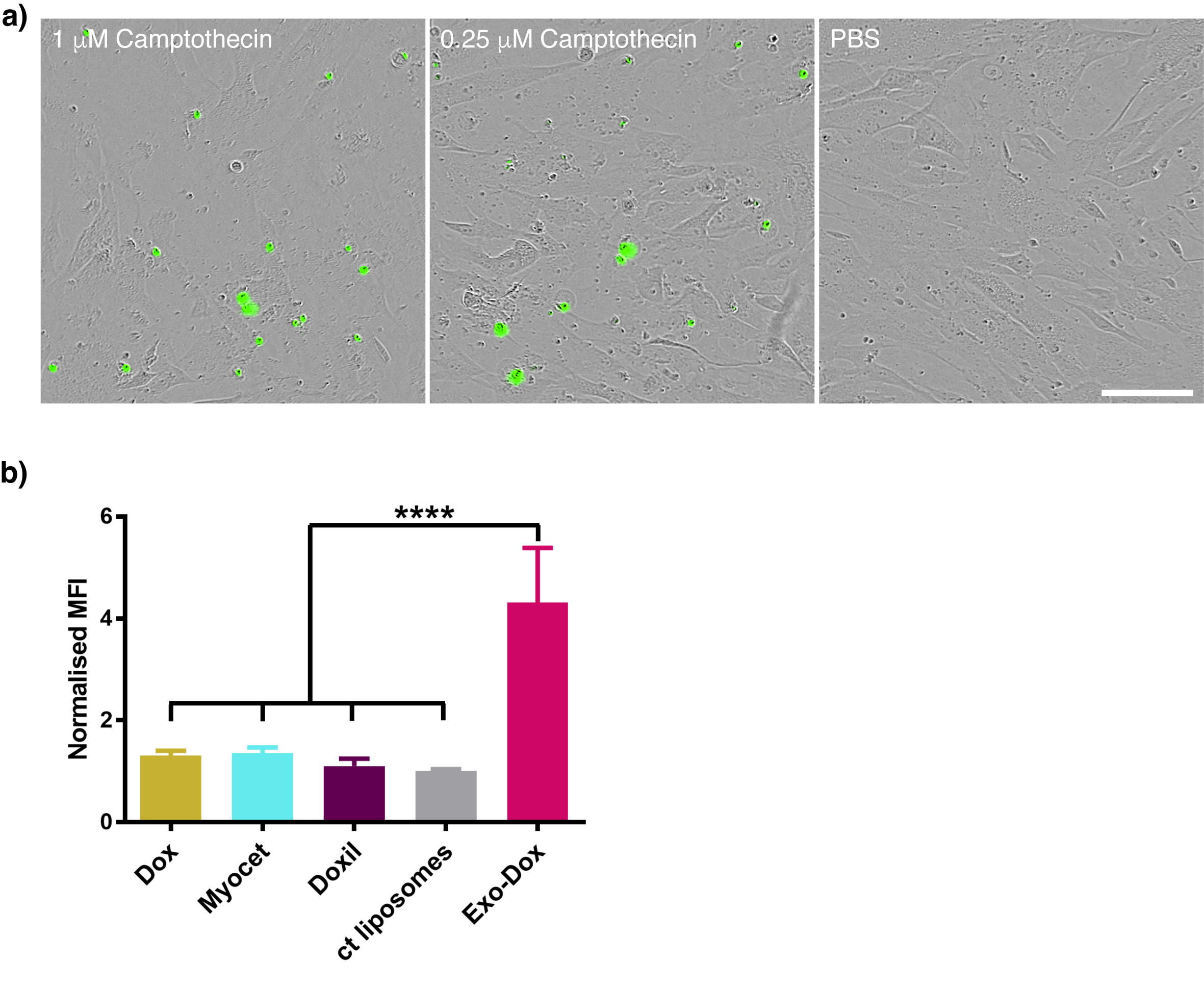

Supplement: S5 Fig — (a) Apoptosis inducer Camptothecin was added to PASMC cells for 24h at concentrations as indicated in the figure in presence of a caspase 3 sensitive fluorogenic substrate, scale bar 100 μm, n = 1. (b) PASMC cells were treated with Dox, Exo-Dox, liposomal formulations of Dox at 0.25 μg/ml for 4 h; uptake was analysed by flow cytometry as described in Fig 1. n = 3, data is displayed as mean +/- SD, ****p<0.0001. (TIFF) [file pone.0214545.s005.tiff]

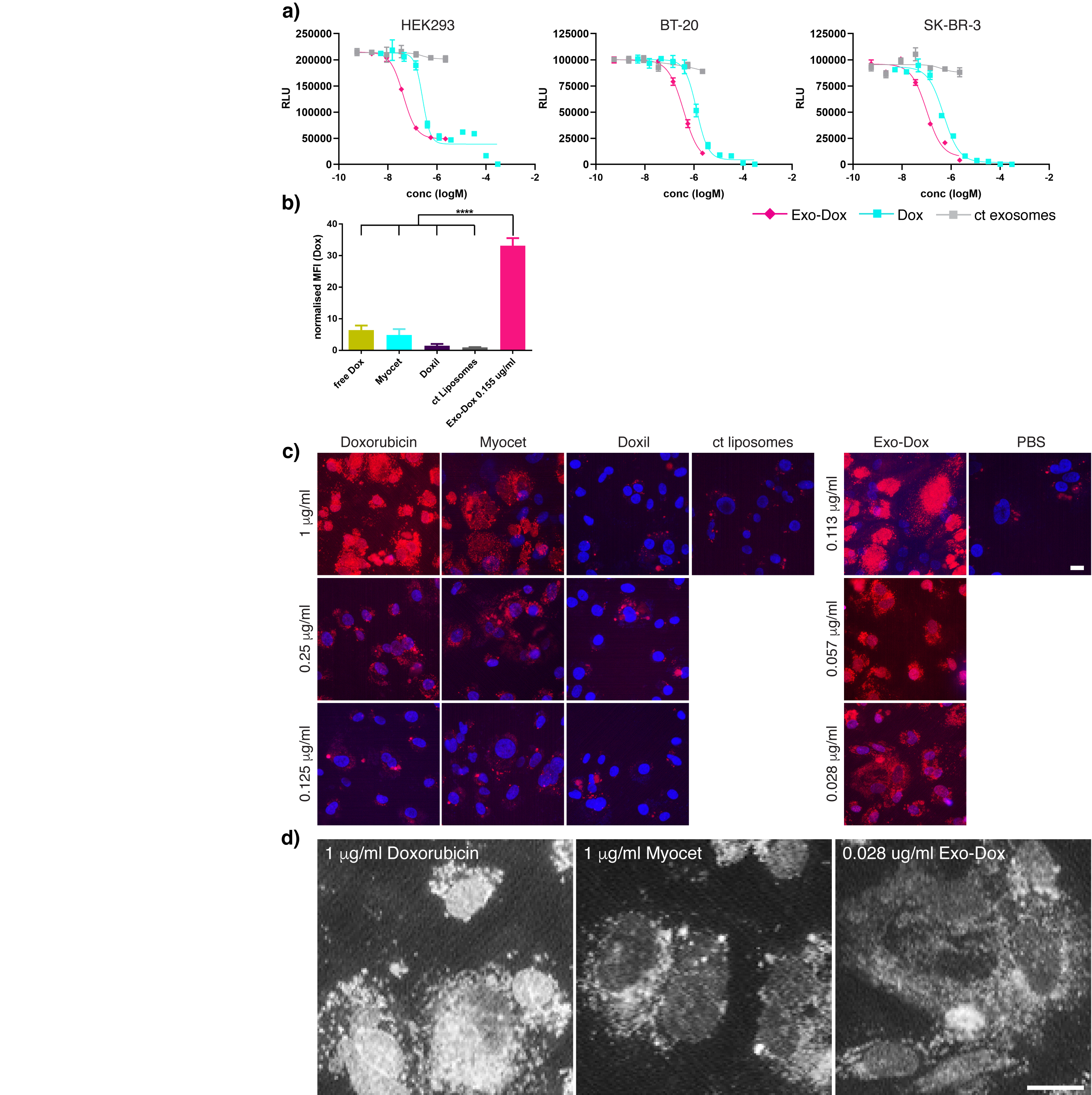

Supplement: S6 Fig — (a) HEK293, BT-20 and SK-BR-3 cells were treated with increasing amounts of free Dox, Exo-Dox or an equivalent particle number of non-loaded control exosomes. Cellular ATP content as measure od viability was determined as in Fig 5; n = 1 data is presented as mean +/- SD. (b) hiPS cardiomyocytes were treated with Dox, Exo-Dox, liposomal formulations of Dox at 0.155 μg/ml for 4 h; uptake was analysed by flow cytometry as described in Fig 1. n = 3, data is displayed as mean +/- SD, ****p<0.0001. (c). hiPS cardiomyocytes cells were incubated with Dox, Exo-Dox, liposomal formulations of Dox (red) at concentrations indicated for 4 h followed by staining of the nuclei with Hoechst (blue). Uptake was analysed by epifluorescence microscopy; representative images from one (out of three) independent experiments are shown. (d) magnified images showing similar red fluorescence intensities from the panel in (c); scale bars: 10 μm. (TIFF) [file pone.0214545.s006.tiff]
