# Supplementary material for: Exosomal delivery of doxorubicin enables rapid cell entry and enhanced in vitro potency
Source: PLoS One. 2019 Mar 29;14(3):e0214545. doi: 10.1371/journal.pone.0214545 (PMC6440694; doi:10.1371/journal.pone.0214545)
Supplement: S1 Methods — (DOCX) [file pone.0214545.s007.docx]

**SUPPLEMENTARY DATA**

**Supplementary experimental procedures**

Analysis of cell viability during prolonged culture in serum-free medium

Adherent HEK293 cells were rinsed twice with PBS and cultured in serum-free Freestyle medium or MEM 10% FBS 1% NEAA over the course of 3 days. Viability was determined by trypan-blue exclusion using automated cell counting (ViCell, Beckman Coulter). 2 T175 flasks were analysed per condition per day (n=2).

Exosome isolation by Ultracentrifugation

Exosome-containing conditioned medium was generated as described in material and methods, likewise cells and large debris were removed by centrifugation and filtration through 0.2 µm filters. The filtrate was processed by centrifugation for 20 min at 10,000xg in an SS34 rotor (Sorvall). Pellets were discarded and supernatants further processed by ultracentrifugation at 110,000xg for 75 min using a TLA-45 rotor (Beckman). Supernatants were discarded and exosome-containing pellets were resuspended in a volume of PBS corresponding to ~1/250 of the original volume.

Generation of total cell extracts, protein concentration determination and Immunoblot

Cells were lysed in RIPA buffer (Sigma-Aldrich) supplemented with a Protease inhibitor cocktail (Sigma-Aldrich) for 5 min on ice. Cellular debris was removed by 10 min centrifugation at 10,000xg. Protein concentration the supernatant as well as in exosomes was determined using a micro-BCA assay (ThermoFisher Scientific) according to manufacturer’s instructions. 10 µg total protein total cell extract or exosomes obtained by either filtration concentration or ultracentrifugation (UC) were resolved on SDS-PAGE gels (Thermo-Fisher Scientific) alongside moleculer mass standard (Li-COR Biosciences), transferred onto nitrocellulose membranes and probed with antibodies to CD63, Alix, Tsg101 and calnexin (all purchased from Abcam; #ab59479, #ab88388, #ab83, #ab22595). Additional antibodies used were to ß-actin (Li-Cor Biosciences, #926-42210) and fibronectin (Sigma-Aldrich, #F3648) as well as fluorescently labelled secondary antibodies (Li-COR Biosciences) for visualisation of bands on an Odyssey imaging system (Li-COR Biosciences).

Co-localisation of Exo-Dox with markers for endocytic pathways

HEK293 cells were co-incubated with 0.26 µg/ml Exo-Dox and 2.5 µg/ml Wheat-Germ-Agglutinin (WGA)-Alexa647 or 50 µg/ml Transferrin-Alexa647 or 1.25 µg/ml Choleratoxin B subunit (CTxB)-Alexa647 or 200 µg/ml Dextran-Alexa647 for 10 minutes at 37 °C in serum-free medium, Hoechst33342 nuclear stain was added at 1 µg/ml and uptake was continued for 5 more min. Cells were washed twice in PBS and switched to culture medium containing FBS for immediate imaging on an Opera confocal imaging system (Perkin Elmer) using a 20x objective and the same exposure settings for all treatments. All Alexa647 labelled tracers and Hoechst33342 were purchased from Thermo-Fisher Scientific.

Exosome uptake in PASMC and iPS cardiomyocytes

Exosome uptake in PASMC and icell cardiomyocytes were perfomed for 4 h as described for HEK293 uptake studies. For PASMC SmBM complete medium and for icell cardiomyocytes the complete medium as provided by the manufacturer was used during the 4 h incubation for uptake.

Timelapse videomicroscopy

Camptothecin (Sigma-Aldrich) was added to HEK293 or PASMC cells at concentrations indicated in the presence of caspase 3 sensitive substrate. Images were acquired every 2 hours for 48h.
